# Lymphoma Heterogeneity: Three Different Histological Pictures and One Unique Clone

**DOI:** 10.1155/2016/3947510

**Published:** 2016-10-27

**Authors:** Sara Alonso-Alvarez, Alba Redondo-Guijo, Óscar Blanco, Miguel Alcoceba, Ana Balanzategui, Juan C. Caballero, Julio Dávila, Marcos González, María D. Caballero, Alejandro Martín, Ramón García-Sanz

**Affiliations:** ^1^Hematology Department, University Hospital of Salamanca, Salamanca, Spain; ^2^Pathology Department, University Hospital of Salamanca, Salamanca, Spain

## Abstract

We report a patient who developed up to three different lymphomas with the same clonal IGH rearrangement. She was first diagnosed of splenic zone marginal lymphoma and relapsed for the first time with Hodgkin lymphoma histology and later with diffuse large B-cell lymphoma histology. Subsequent biopsies and analysis of clonally rearranged IGH genes helped to elucidate the clonal relationship between the three histologies and to confirm a common origin from the three tissue histologies. An integrated diagnosis should always be performed in order to achieve the most accurate diagnosis and be able to choose the best therapeutic options for our patients.

## 1. Introduction 

Hodgkin lymphoma (HL) can occur simultaneously with a variety of B-cell non-Hodgkin lymphomas (composite lymphoma) [1]. It is also well known that indolent lymphomas can transform into more aggressive histologies. However, the sequential occurrence of HL after splenic marginal zone lymphoma (SMZL) and a third consecutive diagnosis of diffuse large B-cell lymphoma (DLBCL) is extremely rare.

The analysis of clonally rearranged IGH genes has helped elucidate the clonal relationship in composite lymphomas involving HL and some cases of sequential development of lymphomas in the same patient. This can also aid a better understanding of tumor biology because it allows the identification of a common origin for theoretically different lymphomas presenting in the same patient.

We report a patient who developed up to three different lymphoma histologies with exactly the same clonal IGH rearrangement.

## 2. Case Presentation

**Figure 1:**
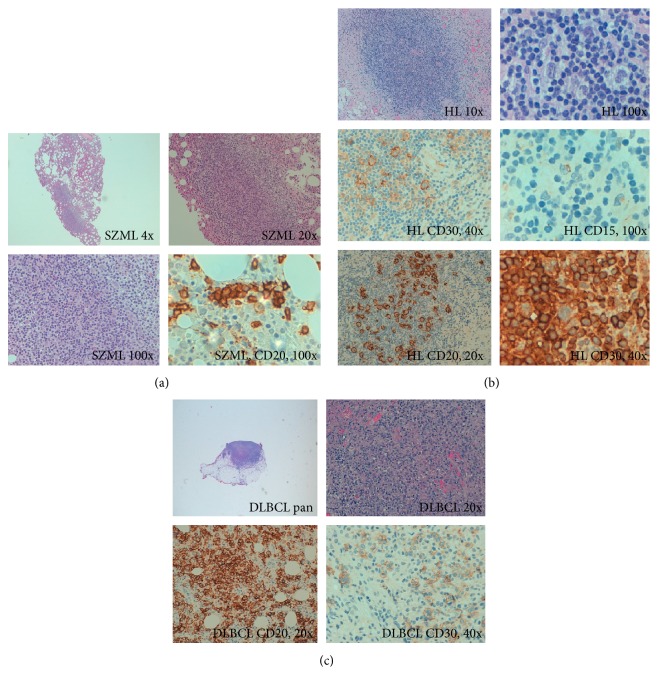
(a) Splenic zone marginal lymphoma. Smz 4x, smz 20x, and smz CD20 100x. Nodular and interstitial bone marrow infiltration by mature small lymphocyte population with a phenotype compatible with splenic marginal zone lymphoma with occasional larger cells (transformed blast cells). With CD20 stain, we observe numerous images of intrasinusoidal infiltration. (b) Nodular sclerosis Hodgkin lymphoma. HL 10x, HL 100x, HL CD30 40x, HL CD15 100x, CD20 20x, and HL CD45 100x. Interaortocaval tumor forms nodules, which are delimited by a hypocellular sclerosing stroma. Each node contains few tumor cells (both Reed-Sternberg and Hodgking type) in an inflammatory background. Tumor cells are CD30+, CD15+ (dot-like staining). CD20+ and CD45−. Morphology and phenotype are diagnostic of classic Hodgkin lymphoma, nodular sclerosis type. (c) Diffuse large B-cell lymphoma. DLBCL pan; DLBCL 20x; DLBCL CD20 20x; DLBCL CD30 40x. Diffuse proliferation of medium/large lymphocytes, mostly with centroblastic habit, which is consistent with DLBCG and which infiltrates adipose tissue. Tumor cells are CD20 positive, with a high proliferative index (Ki67), and non-germinal-center immunophenotype. Inside, we can find occasional CD30+ Sternbergoid cells.

A 66-year-old woman presented in March 2012 with a history of intense fatigue, anorexia, and night sweats that had lasted for two months. Physical examination revealed a giant spleen (maximum diameter 23 cm) and no lymph node enlargement. Her past medical history included only hypertension. Laboratory tests highlighted a small monoclonal IgM kappa component (0.56 g/dL) and anemia (8.6 g/dL). A bone marrow study was diagnostic, since it showed infiltration by monoclonal cells with the following immunohistochemical pattern: CD20+, CD10−, CD5−, CD43−, CD23−, Bcl-2+, Bcl-6−, Cyclin D1−, and MUM.1 focal + (Figure 1(a)), all leading to a diagnosis concordant with splenic B-cell marginal zone lymphoma (SMZL). Flow cytometry (FCM) revealed 28% of tumor cells in the bone marrow with a phenotype compatible with SMZL (CD45+, CD19+, CD5−, CD22+, CD23−/+, CD103−, CD25−, FMC7+, CD11c−, CD20+, CD10−, CD38+, and BCL-2−). A cytogenetic study showed deletion of the long arm of chromosome 7 (which is frequently present in SMZL). She received rituximab treatment (single agent) and achieved complete response after eight courses in July 2013.

**Figure 2:**
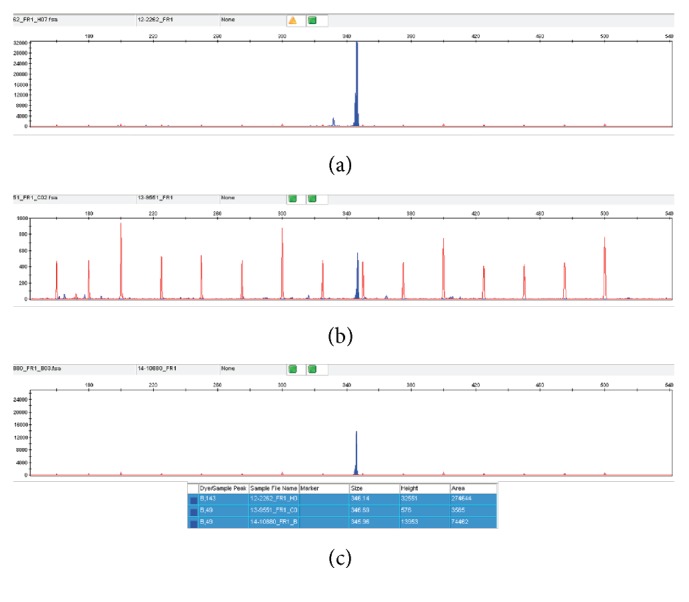
Clonality assessment corresponding to the three histologies. The DNA extracted from the three tumor samples ((a) SZML; (b) HL; (c) DLBCL) was amplified following BIOMED-2 protocol. The clonal peak was detected with FR1 consensus primers. The GeneScan profile obtained from the three histologies showed the same peak of 346 bp. The second sample (corresponding to HL) had the lowest peak.

In October 2013, she presented with a 4-day clinical picture of B symptoms and dyspnea. Physical examination revealed hepatomegaly (3 cm under the costal margin) and splenomegaly (4 cm). A pleural effusion, occupying two-thirds of the left hemithorax, and a left lower lobe consolidation were observed in the chest radiography. Laboratory tests showed an increased LDH (over twice the upper limit of normality) and increased C-reactive protein (10 times the normal level). Liquid obtained from thoracocentesis had exudate characteristics. Effusion cells were revealed to be nonmalignant by cytological examination, and FCM identified them as T cells, with no phenotypic aberrancies. With the diagnosis of left lower lobe pneumonia, without microbiological documentation, she received broad-spectrum antibiotics, to which she responded favorably. After resolution, computed tomography (CT) showed multiple mediastinal, iliac, and retroperitoneal pathological lymphadenopathies. Citrate scintigraphy showed multiple 67Ga pathological deposits distributed throughout the body (in both infraclavicular regions, left armpit, mediastinum, spleen, left iliac chain, dorsal and lumbar spine, pelvis, and right femur). In the absence of significant accessible lymphadenopathies, echoendoscopy was used to guide fine needle aspiration (FNA) in the celiac region. FCM of the FNA sample showed a nonmalignant cell population, mostly comprising T (70%) and B (25%) cells, all without antigenic aberrancies and with a polyclonal distribution. Laparotomy with a lymph node biopsy led to a histological diagnosis of a lymphoproliferative process with large size and Sternberg cells in a T cell environment, forming nodules surrounded by sclerosis. The immunohistochemistry of the large cells was CD45−, CD20+, CD30+, CD15+ (weak), CD3−, BCL6+, MUM.1+, and BCL2−, with a high proliferative index (Ki67) (Figure 1(b)). These features concurred with the diagnosis of nodular sclerosis classic Hodgkin lymphoma. We also investigated IGH clonal rearrangements in the original diagnostic sample from March 2012 (using frozen DNA extracted at diagnosis) and the current HL specimen using consensus primers for the variable and joining segments of the IGH gene, following the BIOMED2 protocol [2]. The PCR product amplicon was evaluated in an ABI Prism 310 genetic analyzer (Applied Biosystems) with GeneScan software (version 3.1.2). We identified the same identical clonal peak in both tumor samples (Figures 2(a) and 2(b)). She received 6 cycles of ABVD (adriamycin, bleomycin, vinblastine, and dacarbazine) and achieved a complete response.

She was observed until August 2014, when fatigue and night sweats started again. A positron emission tomography (PET)/CT scan showed metabolically active disease with supra- and infradiaphragmatic lymph node involvement, highly suggestive of relapsed disease. This time, spleen was not involved. Another excisional biopsy from one of the abdominal lymphadenopathies with intense metabolic activity in PET (SUV = 21.65) showed architectural effacement and extracapsular proliferation. The tumor sample had medium to large lymphocytes, with intense anisonucleosis. CD79 and CD20 staining marked atypical lymphoid elements accompanied by a mature CD5 T lymphocyte population. BCL-2, CD10, BCL-6, and CD30 showed weak, focal positivity. CD45 and MUM.1 were positive in tumor cells, which showed high-grade proliferation with the Ki67 marker. This was concordant with the diagnosis of diffuse large B-cell lymphoma (DLBCL, Figure 1(c)). Once again, the IGH rearrangement evaluation identified a clonal peak that was identical to the one previously seen in the original diagnostic sample (Figure 2(c)). Sanger sequencing identified a highly mutated VDJH sequence (7.1%) using the VH1-2^*∗*^04 and JH6^*∗*^02 genes and the same CDR3 regions in samples obtained in each of the three occasions. With the diagnosis of DLBCL transformed from either HL or SMZL, the patient was treated with 3 courses of R-ESHAP (rituximab, etoposide, cytarabine, cisplatin, and methyl-prednisolone), achieving partial response. After the second cycle, peripheral blood stem cells (PBSC) were mobilized and collected and the patient underwent autologous PBSC transplantation in March 2015 using BEAM (BCNU, etoposide, cytarabine, and melphalan) as conditioning regimen.

Currently, (September 2016) complete metabolic response persists according to the current standard criteria [3].

## 3. Discussion 

This case illustrates the potential intraclonal heterogeneity of tumors. Thus, within the same patient, the same tumor can evolve through different histologies. In addition, we draw attention to the importance of an integrated interpretation of tumor biology given by the combination of clinical, pathological, cytological, and molecular features. High-throughput sequencing recently highlighted the concept of intraclonal heterogeneity in lymphoid tumors [4]. This explains why patients can exhibit different clinical, histological, and biological features during disease evolution [5, 6]. Previous reports have shown a common clonal origin in patients who develop various lymphomas as different as marginal zone lymphoma and Hodgkin lymphoma can be [1, 7–9]. In cases of histologically proved transformations, tumor evolution can show different patterns of behaviour, following either a linear or a divergent pathway from a single mutated precursor [5, 6]. SMZLs are thought to derive from marginal zone cells of the lymphoid follicle, while the Reed-Sternberg (RS) Hodgkin's cells seem to originate from cells that have passed through the germinal center. For DLBCL, we have to differentiate between those with a germinal center origin (GCB) from the activated B cells (ABC) DLBCL, which probably arise from a postgerminal center B-cell arrested during plasmatic differentiation [10].

In our case, all three tumors had a related origin, showing an identical IGH rearrangement. The analysis of the VDJ segment, which was highly mutated, led us to hypothesize that the tumor precursor cell had passed through the germinal center, undergone somatic hypermutation, and acquired ongoing mutations. VH1-2^*∗*^04, the IGVH gene used in this case, was documented in the three tumors, and so it was the same specific CDR3 region. In addition, in the DLBCL sample, we noted small variations in the SHM pattern that indicated the existence of ongoing mutations. It is well known that the VH1-2^*∗*^04 gene is used preferentially in SMZL. In fact, 30% of SMZLs show a VDJH bias towards using this gene. It has recently been shown that they are associated with alterations of chromosome 7q, as was determined in our patient [11]. In Spain, VH1-02 restriction in SMZL accounts for 51% of cases [12], and SMZL with VH1-02 gene rearrangement produces polyreactive antibodies that react against self-antigens; this restricted usage of VH genes and CDR3 regions suggest antigen-driven selection [13]. On the basis of this characteristic IGVH usage and the clinical outcome of the patient, we hypothesize that the original tumor might have been the SMZL. The other two histologies could have developed by transformation of the original tumor.

Current evidence suggests that SMZL, HL, and DLBCL should be treated according to their corresponding therapeutic strategies, irrespective of whether they share the same clonal rearrangement. In our case, we treated the SMLZ with Rituximab, the HL with ABVD, and the DLBCL with salvage therapy consisting of high-dose chemotherapy followed by autologous stem cell transplantation, which are conventional therapies for each condition.

This example reinforces the importance of conducting subsequent biopsies in patients with presumed relapses of low-grade lymphoma, in order to understand the biology of the tumor in each case and, more importantly, to use this knowledge to design and select the best treatment at any moment.
